# Attention-Guided Track-Pulse-Sequence Target Association Network

**DOI:** 10.3390/s26030774

**Published:** 2026-01-23

**Authors:** Yiyun Hu, Wenjuan Ren, Yixin Zuo, Zhanpeng Yang

**Affiliations:** 1 Aerospace Information Research Institute, Chinese Academy of Sciences, Beijing 100094, China; huyiyun23@mails.ucas.ac.cn (Y.H.); zuoyx@aircas.ac.cn (Y.Z.); yangzp@aircas.ac.cn (Z.Y.); 2 Research Department of Cyber-electromagnetic Space Information Technology, Aerospace Information Research Institute, Beijing 100094, China; 3 School of Electronic, Electrical and Communication Engineering, University of Chinese Academy of Sciences, Beijing 100094, China

**Keywords:** target association, satellite electronic information, pulse descriptive word, track segment association

## Abstract

Multi-satellite sequential detection is crucial for maritime target identification and tracking. However, inherent satellite revisit patterns and maritime target motion often result in fragmented track segments, necessitating effective multi-satellite track association to ensure continuity. Existing methods predominantly rely on track information and statistical signal parameters, rendering them susceptible to localization errors and ineffective in scenarios characterized by dense targets and overlapping radar parameters. To overcome these limitations, this paper proposes an attention-guided track-pulse-sequence target association network (AG-TPS-TAN). First, the asymmetric dual-branch network operates by incorporating both track data and electromagnetic signal data, processing the latter in the form of raw pulse sequences instead of the conventional statistical parameters. Second, within the track branch, we enhance the feature representation by incorporating a novel track-point-aware attention mechanism which can autonomously identify and weight critical points indicative of motion continuity, such as interruption boundaries and maneuvering points. Third, we introduce a dual-feature fusion module optimized with a combined loss function, which pulls feature representations of the same target closer together while pushing apart those from different targets, thereby enhancing both feature consistency and discriminability. Experiments were conducted on a public AIS trajectory dataset, constructing a dataset containing both motion trajectories and electromagnetic signals. Evaluations under varying target numbers showed that the proposed AG-TPS-TAN achieved average association accuracies of 93.91% for 5 targets and 63.83% for 50 targets. Against this, the track-only method TSADCNN scored 76.08% and 25.64%, and the signal-statistics-based method scored 77.12% and 29.56%, for 5 and 50 targets, respectively, thus exhibiting a clear advantage for the proposed approach.

## 1. Introduction

Maritime target monitoring holds significant importance in both military and civilian fields. Ship target association and tracking serve as the foundation for realizing maritime target monitoring. Spaceborne electronic reconnaissance, characterized by wide coverage, all-weather, and all-time reconnaissance capabilities, is less susceptible to weather factors and stands as one of the most effective methods for maritime target monitoring. Electronic reconnaissance satellites passively detect and collect various electromagnetic radiation signals from radar systems, communication systems, and weapon telemetry systems. After signal processing, the geographical locations of radiation sources are calculated.

Compared with single-satellite reconnaissance, multi-satellite sequential reconnaissance offers significant improvements in coverage across the time domain, spatial domain, and frequency domain. Multi-satellite sequential reconnaissance can be categorized into successive reconnaissance and common-view reconnaissance. For multi-satellite successive reconnaissance, there exists a certain revisit interval between different satellite reconnaissance passes over the same area, leading to discontinuities in ship tracks. Thus, it is necessary to utilize the electronic information obtained from multi-satellite successive reconnaissance for target association and tracking.

Satellite electronic information comprises target electromagnetic signal information and target motion track information. Electromagnetic signals primarily refer to radar signals, with common parameters including radio frequency (RF), pulse repetition interval (PRI), and pulse width (PW). Motion track information includes longitude, latitude, speed, and course. Based on the types of information utilized, target association can be divided into three major categories: track segment association (TSA), association using electromagnetic signal information, and association combining both track information and electromagnetic information.

Traditional TSA methods rely on prior information and the hypothetical target motion models. They perform forward prediction and backward retrodiction on track segments and achieve association by leveraging the similarity of predicted and retrodicted values. However, these methods suffer from drawbacks such as unreasonable hypotheses, inapplicable models, and uncertain thresholds [1]. To adapt to dynamically changing environments and complex motion patterns, ref. [1] proposed a track segment association dual contrast neural network (TSADCNN). By utilizing metric learning to learn the feature representations of track segments, TSADCNN simplifies the TSA model and reduces association time.

Nevertheless, for target association tasks in multi-satellite successive reconnaissance, methods that solely use track information are easily affected by the positioning accuracy of spaceborne passive detection systems. Additionally, their association performance is poor in scenarios with dense ship targets. To address these issues and fully utilize electronic reconnaissance information, target association methods based on both track information and electromagnetic information have been proposed. Most of these methods only utilize electromagnetic information at the statistical feature level, failing to fully exploit the temporal information, distribution characteristics, and multi-parameter joint variation information of pulse data. As a result, they are only suitable for traditional radars with relatively fixed signal parameters.

With the development of radar technology, most current ship targets are equipped with multifunction radars. These multifunction radars feature complex modulation and flexible operating modes, and there is overlap in statistical parameters among different radar types. Consequently, the aforementioned methods exhibit poor association performance in scenarios where targets are dense and statistical parameters of multiple radar types overlap.

To address the aforementioned issues, we propose AG-TPS-TAN, which performs target association using track information and pulse information in the form of raw pulse sequences rather than statistical parameters. This data-level enhancement provides greater robustness in scenarios involving overlapping multifunction radar parameters. Leveraging deep learning methods to fully excavate the complex features of track data and pulse data, we design corresponding feature extraction networks for track data and pulse sequences, respectively: the former incorporates a track-point-aware attention layer that enables the model to dynamically identify and emphasize critical points, while the latter employs a hierarchical architecture comprising dilated convolutions, a bidirectional LSTM, and a common feature aggregation layer that enhances the stable features shared across multiple pulse trains from the same target. This asymmetric dual-branch architecture ultimately generates a highly discriminative representation that robustly characterizes both the motion patterns and intrinsic signal attributes of each target. The extracted features are then integrated by an effective dual-feature fusion module, which is designed to synthesize complementary information from both track and pulse data, rather than allowing interference. Based on these fused features, we conduct target association. This approach breaks through the limitations of relying on a single type of information, solves the problem of poor association performance of existing methods in scenarios with dense targets and overlapping statistical parameters of multiple radar types, and improves the target association accuracy in complex scenarios.

The rest of this paper is organized as follows. Section 2 reviews the related work. Section 3 introduces our algorithm in detail. Section 4 shows our experiments and performance. Finally, we conclude our work in Section 5.

## 2. Related Work

Target association methods based on track information can be categorized into traditional methods and neural network-based methods. Traditional methods are further divided into three types: statistical methods, fuzzy logic methods, and filtering methods. Statistical methods include the Nearest Neighbor (NN) algorithm, Global Nearest Neighbor (GNN) algorithm, Multiple Hypothesis Tracking (MHT) algorithm, Probabilistic Data Association (PDA) algorithm, Joint Probabilistic Data Association (JPDA) algorithm, and Probabilistic Multiple Hypothesis Tracking (PMHT) algorithm, among others. For fuzzy logic methods, the study in [2] integrates fuzzy logic into the interacting multiple-model probabilistic data association method, significantly reducing target association errors; ref. [3] employs fuzzy track similarity measurement to perform association by leveraging the similarity between track segments. Filtering methods leverage techniques such as Kalman filtering, particle filtering [4], and the Interacting Multiple Model (IMM) [5] to perform forward prediction and backward retrodiction on track segments and then associate targets by using the similarity between predicted and retrodicted values. Ref. [6] first proposed the concept of interrupted track association. It realizes the association of interrupted tracks by extrapolating two track segments to the estimated intersection time and then using a statistical test method based on the maximum likelihood ratio. Ref. [5] introduced the fine-step Interacting Multiple Model (IMM) into the interrupted track association task and formulated the multi-target track segment association problem as a globally constrained optimization problem. To address the issue of track interruption in Ground Moving Target Indication (GMTI) radar tracking, which is caused by targets adopting evasive “move–stop–move” maneuver patterns, ref. [7] proposed an Interacting Multiple Model estimator with State-Dependent Transition Probabilities (IMM-SDP) to predict and retrodict the motion states of track segments. Ref. [8] proposed storing track segment data as a track graph, where nodes represent track segments and edges represent potential associations between track segments. This method transforms the track segment association problem into a minimum-cost flow problem and exhibits better scalability and computational efficiency when handling long-term tracking tasks in high-density environments.

The aforementioned traditional methods rely on prior information and assumed target motion models and suffer from drawbacks such as unreasonable assumptions, inapplicable models, and uncertain thresholds, making it difficult for them to adapt to dynamically changing environments and complex motion patterns. With the development of neural networks, researchers have begun to apply them to interrupted track association tasks. One approach performs point-by-point forward prediction and backward retrodiction on track segments, followed by track association. This type of model is complex and requires a long association time. The other approach leverages contrastive neural networks to extract features from track segments and determines whether two track segments are associated by calculating the similarity between their feature vectors. Contrastive neural networks refer to a category of models centered on the core idea of “contrastive learning”. Their primary goal is to learn to distinguish between “similar sample pairs” and “dissimilar sample pairs”. By reducing the distance between feature vectors of similar objects and increasing the distance between feature vectors of dissimilar objects, contrastive neural networks can strengthen the semantic correlation between similar samples in the feature space and ultimately generate more robust and discriminative feature representations.

Xiong et al. introduced neural network methods into TSA and proposed a TSA dual contrastive neural network (TSADCNN) [1]. That model used a neural network to extract feature representations of track segments, bringing track segments belonging to the same target closer in the high-dimensional space while pushing those belonging to different targets farther apart. Finally, track association was performed in accordance with the nearest-neighbor rule. Ref. [9] converted interrupted tracks into track situation maps and leveraged a generative adversarial network to directly generate continuous tracks. Since simply extracting track position information to form track vectors in traditional methods leads to the loss of irregular structural information, ref. [10] proposed a Track Graph Representation Association (TGRA) method. That method acquired representations of track segments through node-level local track-point embedding and graph-level track-graph embedding, while preserving irregular structural information.

Beyond these, several other methods have been proposed. Ref. [11] develops a Multi-Sensor Track Correlation algorithm based on a Deep Siamese Network for track association and a Multi-Sensor Track Segmentation Association algorithm based on a Deep Comparison Network for interrupted track association via deep learning-based feature extraction and contrastive optimization. Ref. [12] proposes an unsupervised method using homography estimation to estimate radar bias and achieve track association without labeled pairs. Ref. [13] implements interrupted track association through K-step hypothesis testing combined with initial motion constraint screening. Ref. [14] designs the spatial-temporal attention network with spatial–temporal attention modules to capture track dependencies and predict association probabilities end-to-end. Ref. [15] proposes a network utilizing contrastive learning and the Transformer encoder and a two-stage online algorithm for unified interrupted and multi-source track association. Ref. [16] proposes a support vector machine and generative adversarial network hybrid algorithm to associate interrupted tracks via acoustic features and realize track continuation.

In the field of spaceborne electronic reconnaissance, besides track information, target association can also be performed based on electromagnetic information. Traditional radar signal association and matching often employ clustering methods, including K-means clustering, hierarchical clustering, fuzzy clustering, and grid clustering, among others [17]. To address radar signal ambiguity caused by complex environments and counter-detection measures, Zhang et al. [18] introduced a fuzzy pattern recognition method by establishing a fuzzy function to calculate the confidence level between the radar to be identified and known template radars. Guan et al. [19], leveraging the concept of dynamic programming, designed a PRI periodic signal association and batch integration algorithm to tackle the issue of batch increase resulting from the multi-value nature of RF and PW. Wang et al. [20] determine whether two signals come from the same target by checking if the ranges of PRI, PW, and RF parameters of Pulse Description Word (PDW) streams match. Essentially, that method still relies on the statistical features of electromagnetic information and is not applicable to complex and variable multifunction radars. Zhang et al. [21] proposed a PDW homology matching algorithm based on the self-attention mechanism, which excavated the temporal information of pulse sequences. However, their algorithm assumed that the PDW modulation type of the same target was unique, and it was relatively cumbersome as different matching networks needed to be trained depending on whether the data were agile.

To fully utilize satellite electronic information and achieve high-precision ship target association in complex scenarios—such as the scenario of dense targets and overlapped radar parameters—it is natural to leverage both track information and electromagnetic information. Bar-Shalom et al. simultaneously used target class information and target kinematic information for target tracking, incorporating the classification results into a multi-dimensional likelihood [22]. Ding et al. introduced electromagnetic parameter information into the formulation of tracking gate rules and the calculation of association probabilities within traditional passive tracking algorithms [23]. Experts, including Wang Jiegui and Luo Jingqing, integrated target electromagnetic signal information into multi-target association and tracking using Dempster–Shafer evidence theory and gray relational analysis [24,25]. Li et al. [26] utilized target feature information, such as target amplitude information and target Doppler information, for auxiliary association. Liu et al. [27] proposed a track association algorithm assisted by detection point distribution features. That algorithm extracted the distribution features of target detection points using a standard deviation ellipse and performed association by combining state information and monitoring point distribution features, thereby improving the target association accuracy of high-range resolution radars. Liu et al. [28] proposed that on the basis of rough association of target tracks, fine-grained target association should be implemented by maximizing the joint probability distribution of optical/electrical features. Ref. [29] utilized signal attribute features and motion pattern features to judge the similarity of stable track segment pairs from two perspectives: “motion consistency” and “signal attribute consistency”, thus reducing the possibility of merging track segments of different targets.

However, existing target association methods that combine track information and electromagnetic signal information still rely on the statistical features of radar signals and fail to fully excavate the temporal information of signals. Given the widespread application of multifunction radars with complex modulation and flexible working modes, these target association methods have suboptimal performance. To overcome this limitation, a promising direction is to utilize raw pulse sequences directly. Yet, adopting pulse sequences introduces a new core challenge: how to effectively fuse the heterogeneous yet complementary information from the temporal signal stream and the kinematic track data. Without a dedicated fusion mechanism designed for this cross-modal interaction, the potential gains from using rich pulse data may not be fully realized.

## 3. Methodology

**Figure 1:**
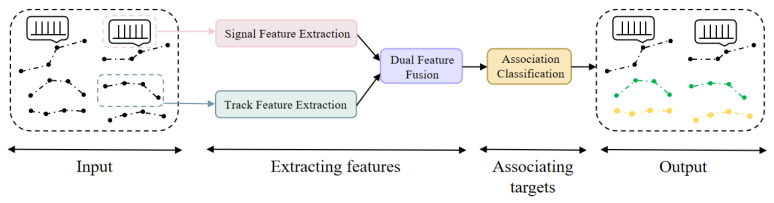
The overall structure of AG-TPS-TAN.

The overall structure of AG-TPS-TAN is shown in Figure 1. Designed to leverage the complementary nature of kinematic and electromagnetic data, the network adopts an asymmetric dual-branch architecture followed by a dedicated fusion module. This design addresses two key challenges: (1) learning effective representations from heterogeneous data types (track segments vs. raw pulse sequences), and (2) integrating them into a discriminative feature space for robust association. Specifically, the track branch and the signal branch extract modality-specific features in parallel. Their outputs are then fused by a dual-feature fusion module that explicitly models their interactions, generating a unified representation for each target. Finally, a nearest-neighbor classifier associates targets across different reconnaissance passes based on the cosine similarity of their fused feature vectors. The following subsections detail each component.

In this section, we introduce the architecture of the proposed method in detail. Specifically, we formulate the problem of target association in multi-satellite successive reconnaissance in Section 3.1 and present the data preprocessing method (Section 3.2), detailed network architecture of AG-TPS-TAN (Section 3.3), and our loss function based on Information Noise-Contrastive Estimation (InfoNCE) (Section 3.4).

### 3.1. Problem Formulation

We utilize both track data and electromagnetic signal data for multi-satellite successive reconnaissance target association. The track data include four parameters: longitude, latitude, speed, and course. The electromagnetic signal data, in the form of pulse sequences, comprise three parameters: PRI, PW, and RF. The notations for data acquired from targets during the first and second reconnaissance passes are as follows.

The first reconnaissance pass is defined as:

(1) $$O_{f}^{i}=\left\{o_{1}^{i},o_{2}^{i},\ldots ,o_{m}^{i}\right\},\;i=1,\ldots ,I,\;m=1,\ldots ,M$$

The second reconnaissance is defined as:

(2) $$O_{s}^{j}=\left\{o_{1}^{j},o_{2}^{j},\ldots ,o_{m}^{j}\right\},j=1,\ldots ,J,m=1,\ldots ,M$$

where *i* is the $i_{th}$ target detected in the first reconnaissance pass, *j* is the $j_{th}$ target detected in the second reconnaissance pass, *m* is the $m_{th}$ track point of a track segment obtained in one reconnaissance pass, *I* is the number of targets detected in the first reconnaissance pass, *J* is the number of targets detected in the second reconnaissance pass, and *M* is the number of track points of a track segment obtained in one reconnaissance pass. $o_{m}^{i}=\left\{{\hat{T}}_{m}^{i},{\hat{P}}_{m}^{i}\right\}$ indicates track data ${\hat{T}}_{m}^{i}=\left\{lat_{m}^{i},lon_{m}^{i},vel_{m}^{i},cou_{m}^{i}\right\}$ and pulse sequences ${\hat{P}}_{m}^{i}=\left\{{\hat{p}}_{m_{1}}^{i},{\hat{p}}_{m_{2}}^{i},\ldots ,{\hat{p}}_{m_{l}}^{i},l=1,\ldots ,L\right\}$, where ${\hat{p}}_{m_{l}}^{i}=\left\{pri_{m_{l}}^{i},pw_{m_{l}}^{i},rf_{m_{l}}^{i}\right\}$, *L* is the length of the pulse sequence corresponding to a single track point.

Feature vectors are obtained after two feature extraction modules and a feature fusion module have processed the above track and signal data. According to the nearest-neighbor criterion, the targets with the closest cosine distance in the feature space are associated, and the association result is defined as

(3) $$\Phi \left(i,j\right)=\underset{\begin{array}{c} i=1,\ldots ,I\\ j=1,\ldots ,J \end{array}}{\text{arg}\;\text{min}}\left(1-\cos(Z_{i},Z_{j})\right)$$

where $Z_{i}$ and $Z_{j}$ are the feature vectors of the $i_{th}$ target in the first reconnaissance pass and the $j_{th}$ target in the second reconnaissance pass, respectively.

### 3.2. Data Preprocessing

In the original track data and electromagnetic signal data, the value ranges and dimensionalities of the four-dimensional features of the track and the three-dimensional features of the pulse sequence are completely different. Neural networks struggle to adapt to such raw data, requiring preprocessing of the data. We adopt the 0–1 normalization method to preprocess the track data and pulse sequence data, respectively, which can eliminate the influence of dimensionality, accelerate model convergence, improve numerical stability, enhance model generalization ability, and facilitate feature comparison. The min–max (0–1) normalization method is a general and widely used data processing approach. All target data in one scenario are uniformly normalized. The 0–1 normalization formula is as follows:

(4) $$x=\frac{x-x_{\min}}{x_{\max}-x_{\min}}$$

For track data, $x_{\min},x_{\max}=\min\left\{x_{m}^{i},x_{m}^{j}\right\},\max\left\{x_{m}^{i},x_{m}^{j}\right\}$; for pulse sequence data, $x_{\min},x_{\max}=\min\left\{x_{m_{l}}^{i},x_{m_{l}}^{j}\right\},\max\left\{x_{m_{l}}^{i},x_{m_{l}}^{j}\right\}$, where i=1…I, j=1…J, m=1…M, l=1…L.

### 3.3. AG-TPS-TAN

AG-TPS-TAN adopts an asymmetric structure and consists of two parallel feature extraction modules, one dual-feature fusion module, and a nearest-neighbor association module. Firstly, the track data and pulse sequences of all targets obtained through multi-satellite successive reconnaissance are input into the network. The track data are processed by the track feature extraction module to generate track feature vectors, while the pulse sequences are processed by the signal feature extraction module to produce signal feature vectors. These two types of feature vectors have the same dimension; after being processed by the dual-feature fusion module, similarity metrics between targets from the first reconnaissance pass and the second reconnaissance pass are obtained. After all high-dimensional feature vectors of two reconnaissance passes are generated, the nearest-neighbor rule is applied to sequentially select the high-dimensional feature vectors with the smallest cosine distance as the association results.

#### 3.3.1. Track Feature Extraction Module

**Figure 2:**
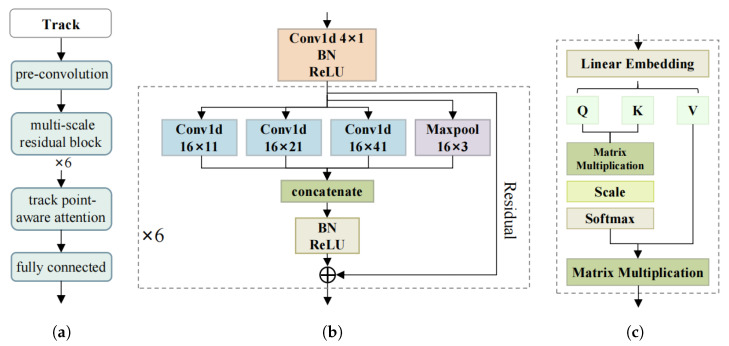
Schematic diagram of the track feature extraction module. (**a**) Overall framework of the track feature extraction module; (**b**) internal structure of the pre-convolution layer and multi-scale residual connection layer, where the orange block represents the pre-convolution layer, and the dashed box denotes the multi-scale residual connection block; (**c**) track-point-aware attention layer.

The track feature extraction module is a dual-branch convolutional neural network. The two branches extract features from the track data of the first and the second reconnaissance pass, respectively. Each branch consists of a pre-convolution layer, a multi-scale residual connection network, and a track-point-aware attention layer. The structure of the track feature extraction module is shown in Figure 2.

Track data exhibit complex and diverse motion patterns. First, a pre-convolution operation is applied to the normalized data to project them into a feature space more amenable to subsequent processing by the multi-scale residual blocks. This process filters noise while preserving the essential fluctuation characteristics of motion information. Then, a multi-scale residual connection network is employed for hierarchical feature extraction. By leveraging convolution kernels of different sizes and maximum pooling operations, this architecture captures discriminative features across multiple temporal scales. Specifically, smaller convolution kernels can capture detailed features such as short-term speed fluctuations, while larger convolution kernels can capture trend features of tracks over longer time spans, thus more comprehensively describing the dynamic changes of tracks. Furthermore, there may be complex nonlinear relationships between the longitude and latitude of successive points within a track segment. The stacked convolutional layers and activation functions in the residual network can effectively capture these nonlinear relationships, and this capability achieves better performance in distinguishing target tracks with complex motion patterns and diverse speed changes.

Critical track points are those that characterize unique target motion patterns and reflect continuity across track interruptions. Based on the motion laws of maritime ships and the characteristics of interrupted track segments, we classify these critical points into three distinct categories. The first category comprises motion trend inflection points, which include turning points and speed mutation points. These points directly reflect target maneuvering behavior through significant changes in course or speed, respectively, and provide stable, distinguishable motion features that aid in differentiating between targets. The second category comprises the boundary points of track interruption, specifically the start and end points of each segment captured in successive satellite passes. These points are crucial for reconnecting discrete segments that originate from the same target. To establish this linkage, the spatiotemporal continuity that must exist between segments of a single target is assessed using the position, speed, and course information at these boundaries, following correction for the intervening time interval. This information forms the core basis for evaluating continuity across segments. The third category is defined by steady motion maintenance points. These are track points where a target sustains a consistent speed and course across multiple consecutive observations. Such points reveal inherent navigation traits, for example, sustained cruising behavior, thereby providing a reference baseline against which the consistency of the target’s motion pattern can be verified before and after a track interruption.

To leverage these three categories of critical points, we implement the track-point-aware attention layer using a self-attention mechanism. This allows the model to dynamically evaluate the importance of each point within the context of the entire sequence and assign correspondingly higher weights to points that are most discriminative for association, such as those revealing motion continuity across interruptions. Let the input to the track-point-aware attention layer be $X=\{x_{1},x_{2},\ldots ,x_{M}\}$, then

(5) $$Q=X\cdot W_{Q},K=X\cdot W_{K},V=X\cdot W_{V}$$

(6) $$Attention\left(Q,K,V\right)=SoftMax\left(\frac{QK^{T}}{\sqrt{d_{k}}}+B\right)\cdot V$$

where *Q* represents the query, *K* represents the key, *V* represents the value, *B* is the position bias, $W_{Q},W_{K},W_{V}$ are learnable weight matrices, and $d_{k}$ denotes the dimension of the key of one attention head, which is used to scale the dot product results to avoid gradient vanishing or explosion. Attention(Q,K,V) is the weighted summation result using attention weights. The output of the track-point-aware attention layer is as follows:

(7) out=X+Attention(Q,K,V)

#### 3.3.2. Signal Feature Extraction Module

**Figure 3:**
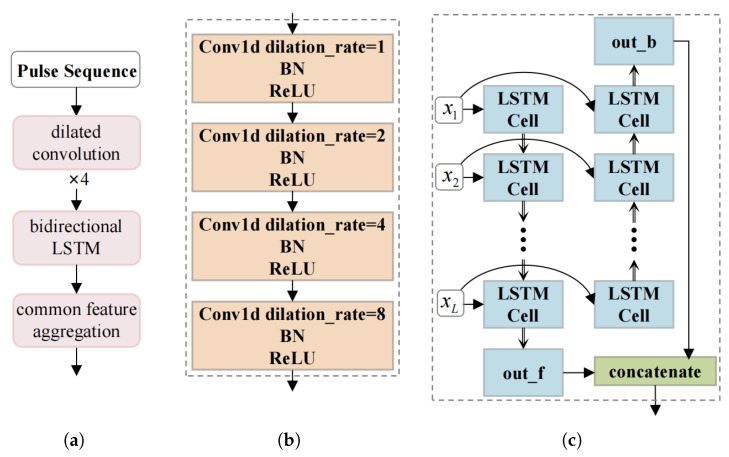
Schematic diagram of the signal feature extraction module. (**a**) Overall framework of the signal feature extraction module; (**b**) dilated convolution layer; (**c**) bidirectional LSTM layer.

The structure of the signal feature extraction module is shown in Figure 3.

The signal feature extraction module is a hybrid network model consisting of two serial networks, which extract local features and sequential temporal features from pulse sequences, respectively. Specifically, the local feature extraction component uses a multi-layer dilated convolution network to hierarchically capture local patterns within the pulse sequences. In the sequential temporal feature extraction component, a bidirectional Long Short-Term Memory (LSTM) is first used to learn the complex temporal dependencies between each pulse and its contextual predecessors and successors; then, a fully connected layer is employed to generate a feature vector of each pulse sequence. Suppose that *M* pulse sequences can be obtained from a single reconnaissance operation on a ship target; then, *M* feature vectors can be obtained. These *M* feature vectors are processed through a common feature aggregation layer to generate a common feature vector, which characterizes the radar signal attributes of the specific ship target. This aggregated feature vector can be used to distinguish different targets, thereby fulfilling the objective of target association.

The common feature aggregation layer consists of four sequential steps: stable feature enhancement, feature alignment, convolution aggregation, and dimensional mapping. Let the input sequence to this layer be denoted as *X*, and the dimension of *X* is M×D. *M* represents the number of track points, and *D* is the output dimension of the preceding bidirectional LSTM layer. Stable feature enhancement aims to reinforce the stable features shared across multiple pulse sequences belonging to the same target. It outputs an enhanced feature representation $X_{\mathrm{stable}}=\tanh\left(\mathrm{BatchNorm}(X\cdot W_{s}+b)\right)$. Utilizing a multi-head self-attention mechanism, the feature alignment layer learns attention weights between each position and all other positions in the sequence, thereby capturing long-range contextual dependencies. It produces a globally contextualized feature representation $X_{\mathrm{aligned}}$.

(8) $$O_{i}=\mathrm{softmax}\left(\frac{Q_{i}\cdot K_{i}^{T}}{\sqrt{d_{k}}}\right)\cdot V_{i}$$

(9) $$X_{\mathrm{aligned}}=\mathrm{concat}\left(O_{1},O_{2},\ldots ,O_{h}\right)\cdot W_{o}$$

where *i* is the index of the attention head, *h* is the number of attention heads, $Q_{i}=X\cdot W_{Q}^{i},\;K_{i}=X\cdot W_{K}^{i},\;V_{i}=X\cdot W_{V}^{i}$, and $W_{Q}^{i},\;W_{K}^{i},\;W_{V}^{i},\;W_{o}$ are learnable weight matrices.

The convolution aggregation layer is designed to capture similarities across multiple pulse sequences from the same target, thereby emphasizing stable and consistent features. This layer applies multi-layer convolutional operations to $X_{\mathrm{aligned}}$. Let RBC(X)=ReLu(BatchNorm(Conv2d(X))); then, the output of that layer is $X_{ca}=RBC\left(RBC\left(RBC\left(X_{wa}\right)\right)\right)$.

#### 3.3.3. Dual-Feature Fusion Module

**Figure 4:**
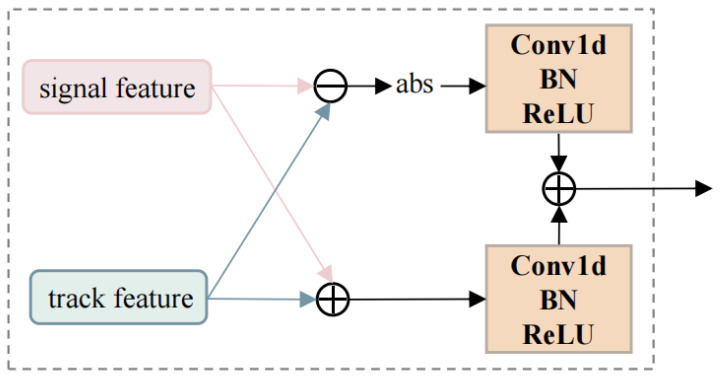
Schematic diagram of the dual-feature fusion module.

The Dense Fusion Module (DFM) [30] was proposed for bi-temporal change detection in Siamese networks, enabling more robust fusion of bi-temporal features. We made adaptive modifications to the DFM for fusing track features and signal features and hence call it the dual-feature fusion module. The schematic diagram of the dual-feature fusion module is shown in Figure 4.

The dual-feature fusion module consists of two branches: The sum branch integrates signal features and track features through element-wise addition. This operation comprehensively combines information from both feature vectors, preserving and enhancing their common characteristics, thereby highlighting the target’s consistent manifestations in both track behavior and signal attributes. The difference branch generates variation-aware features through element-wise subtraction. This emphasizes discrepancies between the two feature vectors, capturing distinctive information that reflects differences in targets’ track motion and signal characteristics. The feature fusion module can organically fuse track features and signal features, making full use of their complementary information, thus achieving better association results than using a single data modality. Let $f_{t}$, $f_{p}$ denote the track feature and the signal feature, respectively. Then, the outputs of the sum branch and difference branch are as follows:

(10) $$O_{s}=f_{t}+f_{p}$$

(11) $$O_{d}=\mathrm{abs}\left(f_{t}-f_{p}\right)$$

The track data and pulse data acquired from the first reconnaissance pass are processed through the aforementioned three modules to obtain a set of feature vectors, denoted as $F_{1}=\left\{f_{1}^{1},f_{2}^{1},\ldots ,f_{I}^{1}\right\}$, where *I* represents the number of targets. Similarly, the feature vectors derived from the second reconnaissance pass are denoted as $F_{2}=\left\{f_{1}^{2},f_{2}^{2},\ldots ,f_{J}^{2}\right\}$, with *J* being the number of targets. To measure the similarity between targets across the two passes, the cosine distance $d_{ij}$ between every pair of feature vectors from $f_{i}$ and $f_{j}$ is calculated as follows:

(12) $$d_{ij}=1-\cos\left(f_{i}^{1},f_{j}^{2}\right)$$

Then, a cosine distance matrix $D_{cos}$ is calculated as follows:

(13) $$D_{\cos}=\left[\begin{array}{ccc} d_{11} & \ldots & d_{1J}\\ \ldots & \ldots & \ldots \\ d_{I1} & \ldots & d_{JJ} \end{array}\right]$$

Our association classification module employs a nearest-neighbor approach, where two targets with the smallest distance in $D_{cos}$ are predicted as associated targets.

### 3.4. Loss Function

To train the aforementioned network, an appropriate loss function must be adopted to optimize the network parameters. The objective is to ensure that the feature vectors extracted by AG-TPS-TAN meet the following requirements: the cosine distance between feature vectors of the same target obtained from two reconnaissance passes should be minimized, while the cosine distance between vectors of different targets should be maximized. The loss function is described as follows:

(14) $$L=L_{T}+L_{P}+\lambda \cdot L_{TP}$$

where $L_{T}$ denotes the loss of the track feature extraction module, $L_{P}$ represents the loss of the signal feature extraction module, and $L_{T}P$ corresponds to the loss of the dual-feature fusion module. In our framework, λ is a key hyperparameter that weights the loss of the dual-feature fusion module. Theoretically, λ can be any positive real number (λ>0), as this loss is essential for learning consistent and discriminative fused features. In this work, we set λ=8. The loss function is implemented using InfoNCE, which is formulated as follows:

(15) $$L_{\left\{T,P,TP\right\}}=-\log\frac{\exp(q\cdot k^{+}/\tau )}{\sum_{k^{+}}\exp\left(q\cdot k^{+}/\tau \right)+\sum_{k^{-}}\exp\left(q\cdot k^{-}/\tau \right)},\;q=\left\{q_{T},q_{P},q_{TP}\right\},\;k=\left\{k_{T},k_{P},k_{TP}\right\}$$

where *q* represents the query vector, $k^{+}$ denotes the positive key vectors, specifically, those from the same target across two reconnaissance passes, and $k^{-}$ signifies the negative key vectors, referring to feature vectors derived from different targets. The temperature parameter τ controls the concentration level of the distribution, influencing how sharply the model focuses on distinguishing between positive and negative pairs. Here, τ is a positive real number. Specifically, $q_{T}$ and $k_{T}$ denote the query and key vectors from the track feature extraction module; $q_{P}$ and $k_{P}$ are from the signal feature extraction module; and $q_{TP}$ and $k_{TP}$ originate from the dual-feature fusion module.

## 4. Experiment

We validated AG-TPS-TAN across scenarios with varying numbers of targets. The experimental results demonstrated that AG-TPS-TAN outperformed other target association methods on experimental datasets. In this section, we start by introducing the evaluation metrics we used. Subsequently, we describe the experimental datasets. Then, we conduct network parameter analysis experiments. After that, we present our results under ideal and non-ideal environments. In the end, we perform a comprehensive set of ablation studies.

### 4.1. Evaluation Metrics

The association accuracy of scenario *n* is defined as follows:

(16) $$acc_{n}=\frac{N_{c}}{N}$$

where $N_{c}$ denotes the number of targets correctly associated, and *N* denotes the number of targets in scenario *n*. The evaluation metric used in this paper was the Average Accuracy (AA) across *S* scenarios, which is defined as follows:

(17) $$AA=\frac{1}{S}\sum_{i=1}^{S}acc_{n}$$

All experiments were conducted in an environment with the PyTorch deep learning framework. The detailed configurations for these experiments were as follows: Ubuntu 22.04, 12 vCPU Intel(R) Xeon(R) Platinum 8352V CPU @ 2.10GHz, vGPU-32GB, PyTorch 2.8.0, Python 3.12, CUDA 12.8.

### 4.2. Dataset

The spaceborne electronic information required in this paper includes track data and electromagnetic signal data. Given the difficulty in acquiring actual satellite electronic reconnaissance data, ship trajectories were simulated using Automatic Identification System (AIS) data. Specifically, this paper adopted the publicly available Multi-source Track Association Dataset (MTAD) [31]; on this basis, electromagnetic parameters were simulated to generate the final dataset. Leveraging this dataset, research on the inter-task target association method was conducted for the data acquired from two electronic reconnaissance tasks.

Radar electromagnetic parameters mainly refer to pulse width (PW), pulse repetition interval (PRI), and pulse frequency (PF); the electromagnetic parameter information is shown in Table 1. Different combinations of modulation types and modulation parameters for electromagnetic parameters, along with different transition probabilities, were selected to simulate the characteristics of electromagnetic parameter variations.

**Figure 5:**
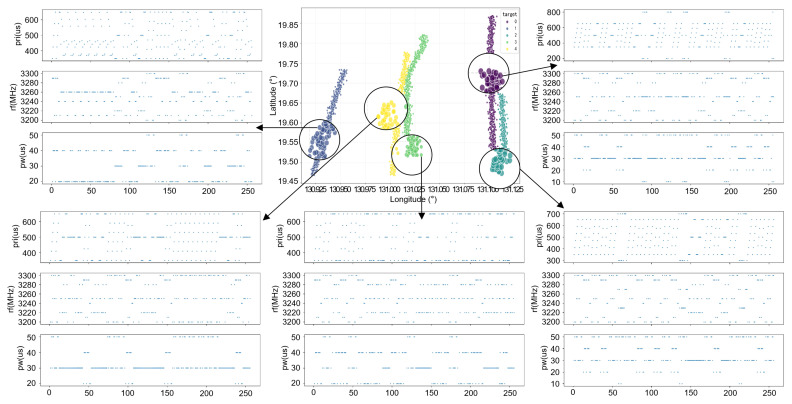
Electronic reconnaissance data.

Taking the scenario with five targets as an example, the final generated data format is shown in Figure 5.

A pair of tracks was defined as the track segments detected in the first and second reconnaissance missions that shared the same label. The training dataset contained approximately 6000 such track pairs, while the validation dataset and test dataset each contained around 750 track pairs.

The radar pulse sequences received in practice are subject to various interferences, leading to non-ideal observation conditions in the pulse sequences, such as measurement errors, lost pulses, and spurious pulses. Meanwhile, the track data also contain certain measurement errors. Gaussian noise with a mean of 0 and standard deviations of 0.02, 0.04, and 0.06 was used to simulate the track measurement errors. The settings of the non-ideal environment in this paper are shown in Table 2.

### 4.3. Network Parameters Analysis

#### 4.3.1. Dimension Analysis of Fused Features

The dimension of the output feature vector exerts a certain impact on the performance of our network. Too small a dimension may fail to accurately characterize the rich information contained in track and signal data, which makes it difficult to distinguish between dense targets with similar pulse sequence parameters or modulation modes. In contrast, an excessively large dimension may introduce redundant information, which not only causes interference during feature fusion but also makes the model harder to converge. To choose the best dimension for subsequent experiments, this paper compared the association performance under different dimensions, with the average association accuracy presented in Table 3.

According to Table 3, with the exception of the scenario involving 10 targets, AG-TPS-TAN obtained the best performance when the dimension of fused features was set to 64 across all other evaluated cases. When the number of targets was 10, a dimension of 64 yielded suboptimal performance. After comprehensive consideration of the results across all scenarios, 64 was selected as the dimension as it provided the best overall balance between representational capacity and generalization.

#### 4.3.2. Temperature Parameter Analysis

The temperature coefficient (τ) is a hyperparameter of the InfoNCE loss function commonly used in contrastive learning. Its core function is to adjust the distribution of feature similarity: τ directly scales the similarity score, and its essence is to control the sharpness of the function output. A smaller τ leads to a steeper output, making the model more sensitive to sample differences, i.e., it focuses more on distinguishing “hard negative samples,” resulting in a finer granularity for judging sample similarity and emphasizing subtle differences. In contrast, a larger τ results in a flatter output, reducing the model’s sensitivity to sample differences; this increases the model’s “tolerance” for negative samples, lowers the discriminability between sample pairs, and shifts the focus from emphasizing subtle differences to prioritizing the overall distribution. To select the optimal temperature coefficient, the average accuracy under different temperature coefficients (0.01, 0.05, 0.07, 0.1, 0.2, 0.3) was compared, and the association performance for different values of τ is shown in Table 4.

According to Table 4, when the temperature coefficient is equal to 0.1, AG-TPS-TAN obtains the best performance.

### 4.4. Association Results Analysis

In order to verify the effectiveness of AG-TPS-TAN, we conducted network adaptability testing, network anti-noise testing, and contrast experiments.

#### 4.4.1. Test Results Under Non-Ideal Observation Conditions

**Figure 6:**
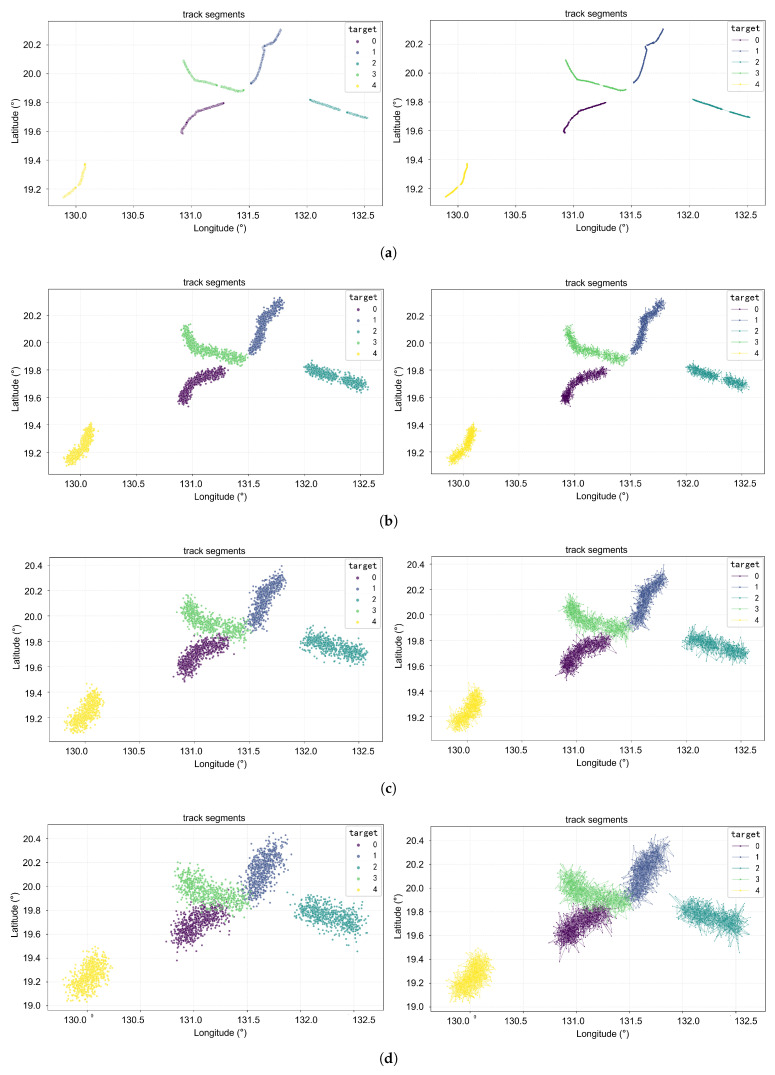
Visual association results under the 5-target condition. (**a**–**d**) Gaussian noise with a mean of 0 and standard deviations of 0, 0.02, 0.04, and 0.06, respectively.

All the aforementioned experiments were conducted based on noise-free datasets. However, non-ideal conditions are prevalent in real-world environments: track data are affected by measurement errors, while pulse sequences suffer from measurement errors, lost pulses, and spurious pulses. To investigate the anti-noise capability of the method proposed in this paper, this section presents test results under non-ideal observation conditions. Three non-ideal observation environments are shown in Table 2, and association results are shown in Table 5. The visual association results of ideal and non-ideal observation environments are shown in Figure 6.

According to Table 5, under challenging conditions where Gaussian noise with a mean of 0 and a standard deviation of 0.06 degrees was added to the track data, and the signal data were corrupted with 1% measurement error, 30% lost pulse, and 30% spurious pulse, the proposed method maintained highly reliable association performance. These results demonstrate that the proposed network possesses strong noise robustness, enabling it to effectively extract meaningful information from both noisy track records and pulse sequences and to successfully integrate them for accurate target association.

#### 4.4.2. Contrast Experiments of Various Association Methods

In this section, our method is compared with traditional TSA (T-TSA) and TSADCNN, which utilize track data. The input data to our network were configured into the following five distinct cases: (i) track data alone, (ii) statistical values of signal parameters (including maximum, minimum, and mean values) alone, (iii) pulse sequence data alone, (iv) a combination of track data and statistical values of signal parameters, and (v) a combination of track data and pulse sequence data. Among these, the combined use of track data and pulse sequence data represents our final proposed method. All contrast experiments were based on the noiseless ideal simulation dataset. The association results of contrast experiments are shown in Table 6.

According to Table 6, the proposed method achieved superior performance across all evaluated scenarios involving varying numbers of targets (5, 10, 15, 20, 30, and 50). Moreover, as the number of targets increased, our approach demonstrated the slowest degradation in association accuracy, highlighting its robustness in high-density environments. Meanwhile, the results further confirmed that leveraging both track information and electromagnetic signal data consistently yielded better association performance compared to using either modality alone. Additionally, for electromagnetic signal utilization, employing pulse sequences led to significantly improved results over methods relying solely on statistical features, underscoring the value of retaining temporal and structural characteristics in the signal representation.

**Figure 7:**
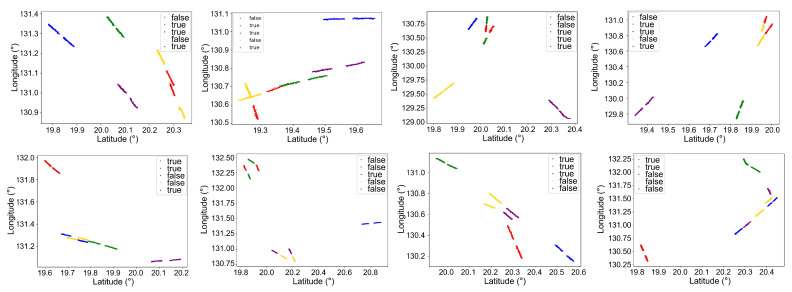
Schematic illustration of misassociation using only track information. Track segments of the same color are associated, indicating they belong to the same target; the labels indicate whether the association is correct.

**Figure 8:**
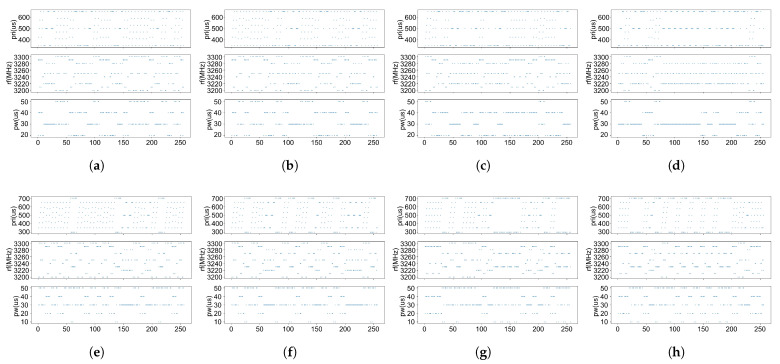
Schematic illustration of misassociation using only electromagnetic signal information. (**a**)-(**b**), (**c**)-(**d**), (**e**)-(**f**), (**g**)-(**h**) represent the two pulse sequences of four targets, respectively. However, they are incorrectly associated as (**a**)-(**d**), (**c**)-(**b**), (**e**)-(**h**), (**g**)-(**f**).

Figure 7 presents the scenarios of association errors when only track information was used. Figure 8 illustrates the instances of association errors when only electromagnetic information was utilized. It can be observed from Figure 7 that when targets are in close proximity, track association using only position information is highly prone to errors. From Figure 8, it is evident that when radar signal parameters overlap or even when radar modulation modes are identical, target association relying solely on electromagnetic signal information also has certain limitations. However, in the scenarios illustrated in the figures, accurate association can be achieved by comprehensively utilizing both track information and electromagnetic information. This result verifies that the proposed method can effectively compensate for the limitations of single-source information, demonstrating that the collaborative fusion of track and electromagnetic information can reliably ensure target association accuracy in scenarios where single-source information fails.

### 4.5. Ablation Study

To verify the effectiveness of the proposed target association network that fuses tracks and pulse sequences, ablation studies were conducted to evaluate the impact of the multi-scale convolution layer, track-point-aware attention layer, common feature aggregation layer, and dual-feature fusion module on association accuracy. All experiments were performed under ideal noise-free conditions, and the association results are shown in Table 7. Herein, “✓” indicates that the module was included, while “×” indicates that the module was removed.

Experiment 1 used our network as the experimental control. Experiment 2 removed the multi-scale convolution layer from the track feature extraction module and replaced it with ordinary one-dimensional convolution layers, which had an equal number of layers to it. Experiment 3 removed the track-point-aware attention layer and directly inputted the output of the previous layer into the subsequent adaptive global average pooling layer. Experiment 4 removed the common feature aggregation layer and replaced it with a fully connected layer, which projected the high-dimensional signal features to the required dimension. Experiment 5 removed the dual-feature fusion module, and the track and signal feature vectors were concatenated and then passed through a fully connected layer to obtain the final feature vector. Experiment 6 removed the above four optimization modules.

According to Table 7, all key components of AG-TPS-TAN—namely, the multi-scale convolutional layer, the track-point-aware attention layer, the common feature aggregation layer, and the dual-feature fusion module—contribute significantly to its overall performance.

## 5. Conclusions

This paper proposed AG-TPS-TAN, a target association network that fuses track segments with raw pulse sequences to overcome the limitations of existing methods in dense target scenarios with overlapping radar parameters. The network features an asymmetric dual-branch architecture: the track branch extracts motion patterns via multi-scale convolution and an attention mechanism, while the signal branch captures temporal features using dilated convolution and a bidirectional LSTM. A dual-feature fusion module integrates these representations, and association is achieved through nearest-neighbor matching in the fused feature space. Evaluations on a dedicated dataset showed that the proposed method significantly outperformed approaches using only track or statistical signal features across varying target densities while maintaining strong robustness under noise. Future work will focus on (1) resolving association ambiguities for targets with identical radar types and highly similar motion patterns and (2) investigating online association mechanisms for streaming multi-satellite observations.

## Figures and Tables

**Table 1:** Electromagnetic parameters setting of targets.

Modulation Parameters		PRI	PW	RF
Initial value interval		(200–1200 μs	(10–50) μs	(3–8) GHz
Fixed	Value	(200–1200) μs	(10–50) μs	(3–8) GHz
Jittered	Range	5–30%	5–30%	5–30%
Staggered	Number of bursts	2–6	2–6	2–6
Agile	Number of bursts	2–6	-	-
Dwell and Switch	Number of bursts	2–4	-	2–4
	Length of the burst	2–6	-	2–6
Sliding	Number of bursts	4–6	-	-
	Deviation	20–100 μs	-	-
Wobulated	Frequency	30–70 Hz	-	-
	Sampling frequency	300–1050 Hz	-	-
	Amplitude	(5–30)%* initial value	-	-

**Table 2:** Non-ideal observation environments.

Environment	Track Noise	Measuring Noise (%)	Lost Pulse(%)	Spurious Pulse (%)
0	0	0	0	0
1	0.02	1	10	10
2	0.04	1	20	20
3	0.06	1	30	30

**Table 3:** Association effects of different dimensions.

Dimension	AA					
	**N = 5**	**N = 10**	**N = 15**	**N = 20**	**N = 30**	**N = 50**
4	0.9214	0.8217	0.7494	0.7144	0.6547	0.6004
8	0.9189	0.8204	0.7529	0.7132	0.6583	0.6215
16	0.9273	0.8301	0.7628	0.7152	0.6694	0.6273
32	0.9232	0.8324	0.7630	0.7202	0.6693	0.6364
64	0.9391	0.8303	0.7654	0.7242	0.6759	0.6383
128	0.9314	0.8231	0.7635	0.7234	0.6732	0.6201
256	0.9271	0.8242	0.7601	0.7175	0.6728	0.6326

**Table 4:** Association effects of different temperature coefficient.

τ	AA					
	**N = 5**	**N = 10**	**N = 15**	**N = 20**	**N = 30**	**N = 50**
0.01	0.9043	0.8054	0.7035	0.6834	0.6259	0.5637
0.05	0.9283	0.8264	0.7476	0.7083	0.6484	0.6035
0.07	0.9364	0.8245	0.7621	0.7189	0.6693	0.6294
0.1	0.9391	0.8303	0.7654	0.7242	0.6759	0.6383
0.2	0.9239	0.8201	0.7463	0.7193	0.6528	0.6201
0.3	0.9256	0.8199	0.7249	0.7002	0.6459	0.6190

**Table 5:** Association results under non-ideal observation environments.

Env	AA					
	**N = 5**	**N = 10**	**N = 15**	**N = 20**	**N = 30**	**N = 50**
1	0.9123	0.8225	0.7534	0.7023	0.6423	0.6095
2	0.9053	0.8034	0.7294	0.6832	0.6248	0.5743
3	0.8832	0.7943	0.7073	0.6548	0.5893	0.5263

**Table 6:** Association results of contrast experiments.

Method	AA					
	**N = 5**	**N = 10**	**N = 15**	**N = 20**	**N = 30**	**N = 50**
T-TSA	0.5033	0.4032	0.3347	0.2921	0.2274	0.1673
TSADCNN	0.7608	0.6481	0.5429	0.4330	0.3546	0.2564
Statics	0.7712	0.6120	0.5067	0.4531	0.3934	0.2956
Track	0.8121	0.7067	0.5648	0.5302	0.4167	0.3240
PulseSeq	0.8991	0.7667	0.6735	0.6302	0.5967	0.5403
Track+Statics	0.8439	0.7324	0.6056	0.5530	0.4627	0.3533
Ours	0.9391	0.8303	0.7654	0.7242	0.6759	0.6383

**Table 7:** Ablation study results.

No.	MSC	TPAAL	CFAL	DFFM	AA					
					**N = 5**	**N = 10**	**N = 15**	**N = 20**	**N = 30**	**N = 50**
1	√	√	√	√	0.9391	0.8303	0.7654	0.7242	0.6759	0.6383
2	×	√	√	√	0.9243	0.8145	0.7394	0.7019	0.6592	0.6069
3	√	×	√	√	0.9254	0.8220	0.7579	0.7125	0.6634	0.6256
4	√	√	×	√	0.9189	0.8196	0.7431	0.7063	0.6593	0.6163
5	√	√	√	×	0.9024	0.8049	0.7402	0.6984	0.6324	0.5998
6	×	×	×	×	0.8103	0.7492	0.6847	0.6531	0.5762	0.4552

## Data Availability

Data are contained within the article.

## Abbreviations

The following abbreviations are used in this manuscript:

AG-TPS-TAN	Attention-guided Track-Pulse-Sequence Target Association Network
LSTM	Long Short-Term Memory
RF	Radio Frequency
PRI	Pulse Repetition Interval
PW	Pulse Width
TSA	Track Segment Association
TSADCNN	Track Segment Association Dual Contrast Neural Network
NN	Nearest Neighbor
GNN	Global Nearest Neighbor
MHT	Multiple Hypothesis Tracking
PDA	Probabilistic Data Association
JPDA	Joint Probabilistic Data Association
PMHT	Probabilistic Multiple Hypothesis Tracking
IMM	Interacting Multiple Model
GMTI	Ground Moving Target Indication
IMM-SDP	Interacting Multiple Models with State-Dependent Transition Probabilities
TGRA	Track Graph Representation Association
PDW	Pulse Description Word
InfoNCE	Information Noise-Contrastive Estimation
DFM	Dense Fusion Module
AA	Average Accuracy
AIS	Automatic Identification System
MTAD	Multi-source Track Association Dataset
T-TSA	Traditional Track Segment Association
MSC	Multi-Scale Convolution Layer
TPAAL	Track-Point-Aware Attention Layer
CFAL	Common Feature Aggregation Layer
DFFM	Dual-Feature Fusion Module
